# The Effect of Chronic NO Synthase Inhibition on the Vasoactive and Structural Properties of Thoracic Aorta, NO Synthase Activity, and Oxidative Stress Biomarkers in Young SHR

**DOI:** 10.1155/2018/2502843

**Published:** 2018-06-28

**Authors:** Andrea Berenyiova, Ima Dovinova, Miroslava Kvandova, Frantisek Kristek, Eugene Jansen, Miroslava Majzunova, Sona Cacanyiova

**Affiliations:** ^1^Institute of Normal and Pathological Physiology, Center of Experimental Medicine, Slovak Academy of Sciences, 813 71 Bratislava, Slovakia; ^2^Centre for Health Protection, National Institute for Public Health and the Environment, 3721 MA Bilthoven, Netherlands

## Abstract

Although the role of nitric oxide (NO) in essential hypertension is still unclear, the effects of long-term NO deficiency have not yet been investigated during the critical juvenile period in spontaneously hypertensive rats (SHR). We aimed to analyze the effects of chronic NO synthase (NOS) inhibition on systolic blood pressure (sBP), vasoactivity, morphological changes and superoxide level in the thoracic aorta (TA), NOS activity in different tissues, and general biomarkers of oxidative stress in plasma of young SHR. Four-week-old SHR were treated with N^G^-nitro-L-arginine methyl ester (L-NAME, 50 mg/kg/day, p.o.) for 4-5 weeks. L-NAME treatment induced a transient sBP increase only, and surprisingly, slightly inhibited endothelium-dependent relaxation of TA. Hereby, the inhibition of NOS activity varied from tissue to tissue, ranging from the lowest in the TA and the kidney to the highest in the brain stem. In spite of an increased sensitivity of adrenergic receptors, the maximal adrenergic contraction of TA was unchanged, which was associated with changes in elastin arrangement and an increase in wall thickness. The production of reactive oxygen species in the TA was increased; however, the level of selected biomarkers of oxidative stress did not change. Our findings proved that the TA of young SHR responded to chronic NO deficiency by the development of adaptive mechanisms on the functional (preserved NO-derived vasorelaxation, unincreased contraction) and molecular (preserved NOS activity) level.

## 1. Introduction

An increase in the systolic blood pressure (sBP) evoked by reduced production of endogenous nitric oxide (NO) after NO synthase (NOS) inhibition has been already considered as a uniform response of the cardiovascular system. In adult normotensive rats, the long-term administration of NOS inhibitors, such as N^G^-nitro-L-arginine methyl ester (L-NAME), elicits an unequivocal pattern of sustained NO-deficient hypertension [1]. Essential hypertension, experimentally represented by spontaneously hypertensive rats (SHR), is characterized by pathological changes similar to alterations developed during NO-deficient hypertension. Increased vasoconstriction and oxidative stress, impaired endothelium-dependent vasorelaxation, and abnormal cardiovascular remodeling accompanied by arterial wall hypertrophy are the main hallmarks of these two experimental models [2]. However, the origin of the abovementioned cardiovascular disorders in SHR is still unclear. The shift of the equilibrium of vasoactive compounds in favor of vasoconstrictors has been suggested as a possible cause of these disorders. Several studies speculated that the cause is a disturbed endogenous NO production and/or the decreased endothelium-derived vasorelaxation in certain arteries within the blood bed [3, 4]. On the other hand, other studies confirmed the potentiation of NO-dependent relaxation associated with an increased NO production by the vessel wall during developed essential hypertension [5, 6]. Nevertheless, oxidative stress is also very important in relation to NO effectivity and/or deficiency. The overproduction of superoxides, found in adult SHR, could lead to the decrease of NO bioavailability [7].

In young prehypertensive SHR, there are still limitations in data about endothelial function and NO signalization and the results are very often conflicting. We have recently demonstrated a widespread participation and abundant effect of NO produced by NOS in the vasoactive responses of the thoracic aorta in 4-week-old prehypertensive SHR [8]. Several other studies confirmed a compensatory role of endogenously produced NO against the increased vascular tone in adult SHR [9, 10]. The chronic experiments with long-term inhibition of NO production could help to specify the role of NO signalization in the etiopathogenesis of essential hypertension. Nevertheless, the effect of long-term NOS inhibition on blood pressure and vasoactivity during the critical juvenile period has not yet been investigated in SHR although this stage is one of the critical periods when the increase in blood pressure could be modified [11]. The aim of this study was to investigate the effect of chronic NOS inhibition using L-NAME in young 4-week-old SHR on sBP and the vasoactive properties of the thoracic aorta (TA). In the TA, we also evaluated wall thickness, elastin arrangement, NO synthase activity, and reactive oxygen species (ROS) formation. We also measured NO synthase activity in the left and right ventricle, kidneys, and brain stem. Reactive oxygen metabolites, total thiols levels, and biological antioxidant potency were measured as selected biomarkers of oxidative stress and antioxidant status in plasma to provide information related to the possible general redox imbalance.

## 2. Materials and Methods

### 2.1. Guide for the Use and Care of Laboratory Animals

Procedures were performed in accordance with institutional guidelines and were approved by the State Veterinary and Food Administration of the Slovak Republic and by Committee on the Ethics of Procedures in Animal, Clinical and others Biomedical Experiments (permit number: EK/noh2s/14) of the Institute of Normal and Pathological Physiology, Slovak Academy of Sciences according to the European Convention for the Protection of Vertebrate Animals used for Experimental and other Scientific Purposes, Directive 2010/63/EU of the European Parliament. All rats used in this study were received from accredited breeding establishment of the Institute of Normal and Pathological Physiology, Slovak Academy of Sciences (permit number: SK U 14016) and were housed under a 12 h light-12 h dark cycle, at a constant humidity (45–65%) and temperature (20–22°C), with free access to standard laboratory rat chow and drinking water. The Institute of Normal and Pathological Physiology provided veterinary care. The health state of the animals was daily monitored and appreciated according to specific criteria used to determine when animals should be euthanized (human endpoint). The features of the body (weight-shortage), physiological functions (food and water intake, disequilibrium, etc.), and social interactions (avoidance reactions, uncoordinated movement, etc.) of the animals were included into these criteria.

### 2.2. Experimental Animals and Treatments

Male 4-week-old SHR were used in the present study. The animals were divided into two groups of 8 animals each. The first group represented the control group (SHR). The second group received L-NAME (50 mg/kg per day) continuously. After four weeks of treatment, we observed the death of one rat as a result of the deterioration of health conditions: disorientation, paralysis of lower extremities, deterioration of the locomotion activity, and the presence of shivers and bouts. The rest of the experimental animals was immediately (at latest in 4 hours) euthanized after the appearance of any abovementioned symptoms. The animals were sacrificed in the 5th week of the treatment by decapitation after brief anesthetization with CO_2_, and all efforts were made to minimize the suffering of the animals. The animals were then used for functional, biochemical, and morphological investigation. The L-NAME uptake was monitored daily according to the respective drink intake. During the experiment, the sBP was measured once a week by the noninvasive plethysmography method.

### 2.3. Physiological Study

The TA was isolated after brief CO_2_ anesthetization and decapitation of the animals. The vessels were cleaned of connective tissue and cut into 5 mm length rings. The rings of the TA were vertically fixed between 2 stainless steel wire triangles and immersed in a 20 ml organ bath filled with Krebs solution (118 mmol/1 NaCl, 5 mmol/l KCl, 25 mmol/l NaHCO_3_, 1.2 mmol/l MgSO_4_.7H_2_O, 1.2 mmol/l KH_2_PO_4_, 2.5 mmol/l CaCl_2_, 11 mmol/l glucose, and 0.032 mmol/l CaNa_2_ EDTA). This solution was oxygenated with a 95% O_2_ and 5% CO_2_ mixture and stored at 37°C. The upper triangles were connected to the sensors of the isometric tension (FSG-01, MDE, Budapest, Hungary), and the changes in tension were registered by an AD converter NI USB-6221 (National Instruments, Austin, Texas, USA) and DEWESoft (Dewetron, Prague, Czech Republic). The resting tension of 1 g was applied to each ring and maintained throughout a 45 to 60 min equilibration period until stress relaxation no longer occurred.

Potassium chloride (100 mmol/l) was added to the organ bath to test the induction of receptor-independent contraction induced by depolarization of the smooth muscle cells. Noradrenaline and phenylephrine were used as activators of *α*-adrenergic receptors of smooth muscle cells. Concentration-dependent contractile responses of the TA were induced by increasing doses of noradrenaline (10^−10^–3 × 10^−5^ mol/l) in a cumulative manner. Contractions were expressed as the developed tension (g) or the percentage of the maximum tissue responses to the agonist (demonstrating the sensitivity to the noradrenaline). The concentrations of noradrenaline that produce the half-maximum response (EC_50_) were calculated from individual dose-response curves and expressed as the negative logarithm of the molar concentration.

The relaxant responses of the TA were followed by phenylephrine-precontracted (10^−6^ mol/l) arterial rings after a stable plateau was achieved. The rings were then exposed to cumulative doses of acetylcholine (10^−10^–3 × 10^−5^ mol/l) or sodium nitroprusside (10^−10^ − 3 × 10^−5^ mol/l). The maximal acetylcholine-induced (10^−5^ mol/l) vasorelaxation of phenylephrine-precontracted (10^−6^ mol/l) arterial rings was also followed before and 20 min after the acute pretreatment with L-NAME (10^−4^ mol/l). The rate of relaxation was expressed as a percentage of the contractile agonist-induced contraction. The concentrations of acetylcholine that produced a 50% inhibition of contraction (IC_50_) were calculated from individual dose-response curves and expressed as the negative logarithm of the molar concentration.

### 2.4. Determination of NO Synthase Activity

Activity of NOS was measured by conversion of radioactive [3H]-L-arginine (Amersham, UK) to [3H]-L-citrulline. The activity of NOS was measured in 20% homogenates prepared in Tris-HCl with the addition of protease inhibitors. The homogenates were centrifuged at 5000 rpm, 10 min at 4°C. Samples were measured in duplicate. The reaction mixture (0.5 M Tris pH 7.4; 10 mmol/l NADPH; 20 mmol/l CaCl_2_ and MgCl_2_; 100 *μ*mol/l L-arginine; 1 mg/ml calmodulin; 1 : 1 FAD/FMN; radiolabeled L-arginine; 50 mmol/l BH_4_; distilled water) and the homogenates (50 *μ*l) were incubated 6 min at 37°C. After incubation, the reaction was initiated by addition of the reaction mixture (50 *μ*l) to the samples. The reaction was stopped after 20 min by the addition of a solution with 1 mmol/l L-citrulline in 0.02 M HEPES, 2 mmol/l EDTA, and 2 mmol/l EGTA. A 1 ml aliquot from the sample was applied to a Dowex column and cycled with 1.5 ml distilled water. Subsequently, the product (samples with ECOLITE scintillation fluid) was detected on the Tri-Carb 2910 TR (PerkinElmer) scintillation counter. NOS activity was expressed as pkat/g of proteins.

### 2.5. Detection of ROS Formation in the Aorta Using Dihydroethidium (DHE) Fluorescent Dye

Aortic ring samples (5 mm) were prepared in Tissue-Tek O.C.T. compound, and cryosections (30 *μ*m) were prepared using a Leica CM1950 cryostat and mounted onto glass slides. Dihydroethidium (DHE, Molecular Probes) oxidation-sensitive fluorescent dye was added to the slides at a final concentration of 10 *μ*mol/l (stock solution: 10 mmol/l in DMSO), and samples were incubated at 37°C for 30 min in the dark as previously described by Oliveira et al. [12]. The production of ROS in the cell nucleus and DHE fluorescence formation was halted by placing the microscope slides on ice. Samples were detected by fluorescence microscopy using a Nikon Eclipse Ti-E and NIS-Elements AR program with excitation 510 nm/emission 595 nm. Quantification of DHE fluorescence intensity was analyzed using ImageJ software.

The measuring of ROS with fluorescent probes could also have some challenges and limitations [13]. In red fluorescence measurements, DHE fluorescent dye can react on two types of products: ethidium (E+) and 2-hydroxyethidium (2-OH-E+). They have similar fluorescent spectra characteristics, and HPLC analysis is required for the proper estimation of superoxide production. The measurements of fluorescent increase due to the oxidation of DHE providing only the general information on redox homeostasis disruption. Nevertheless, fluorescent detection of dihydroethidium oxidative fluorescence microtopography (DHE cryo staining) is a usual technique to assess vascular ROS formation [14].

### 2.6. Determination of Wall Thickness and Elastin Detection

Aortic sections were detected by fluorescence microscopy. Wall thickness (tunica intima + tunica media) of the TA was determined, and the values were measured using fluorescence microscopy.

Native tissue autofluorescence is a widespread phenomenon in which intrinsic biomolecules, such as collagen and elastin in the aorta, act as endogenous fluorophores. The elastin in the TA media was detected by autofluorescence (ex 488 nm/em 510 nm) using a Nikon Eclipse Ti-E fluorescence microscope.

### 2.7. Determination of Plasma Biomarkers

The reactive oxygen metabolites (ROM) assay for hydroperoxides is a spectrophotometric test that determines the concentration of hydroperoxides. The hydroperoxides in plasma react with a chromogenic substrate to develop a colored derivative. The assay (dROMs) was obtained from Diacron International (Grosseto, Italy).

The biological antioxidant potency (BAP) antioxidant assay is a test that determines the total antioxidant status of plasma. The BAP assay is based on the reduction of a colored solution containing ferric ions by antioxidants in plasma. The assay was obtained from Diacron International (Grosseto, Italy).

The total thiol levels (TTL) assay is based on the ability of free thiol groups to develop a colored complex when reacted with 5,5-dithiobis-2-nitrobenzoic acid (DTNB). The color intensity is directly related to the thiol groups which are not affected by oxidation. The assay was obtained from Rel Assay Diagnostics (Gaziantep, Turkey).

All plasma biomarkers were measured using a UniCel DxC 800 (Beckman-Coulter, Woerden, Netherlands) clinical autoanalyzer.

### 2.8. Statistical Analysis

The data were expressed as the means ± S.E.M. For the statistical evaluation of differences between groups, a one-way analysis of variance (ANOVA) with a Bonferroni post hoc test and paired *t*-test was used. The differences between means were considered significant at *p* < 0.05.

### 2.9. Drugs

The following drugs were used for analyses: acetylcholine (Sigma-Aldrich, St Louis, Missouri, USA), N^G^-nitro-L-arginine methyl ester (Sigma-Aldrich), noradrenaline (Zentiva, Czech Republic), phenylephrine (Sigma-Aldrich), potassium chloride (Slavus, Slovak Republic), and sodium nitroprusside (Sigma-Aldrich).

## 3. Results

### 3.1. Systolic Blood Pressure, Biometric Parameters, and Biomarkers of Oxidative Stress

**Figure 1:**
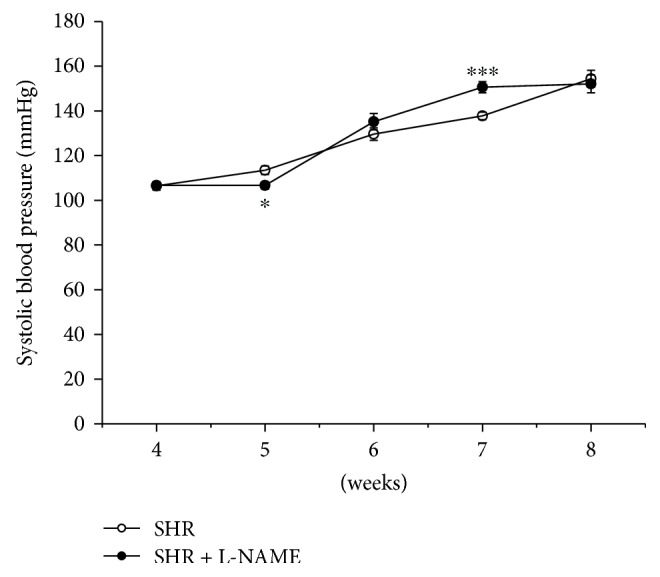
Time course of systolic blood pressure in control SHR and in SHR treated with L-NAME for 4-5 weeks. SHR: control group (*n* = 8); SHR + L-NAME: SHR treated with L-NAME (*n* = 7). Data are expressed as the mean ± S.E.M. ^∗^*p* < 0.01 versus SHR; ^∗∗∗^*p* < 0.001 versus SHR.

Changes in the systolic blood pressure (sBP) were monitored during the experiment in young SHR. The sBP was significantly decreased (SHR: 113.4 ± 1.9 mmHg, SHR + L-NAME: 106.6 ± 1.6 mmHg, *p* < 0.05) after the 1st week and significantly increased (SHR: 137.8 ± 1.7 mmHg, SHR + L-NAME: 150.6 ± 2.5 mmHg, *p* < 0.001) after the 3rd week of L-NAME treatment; however, at the end of the treatment, there were no differences in sBP between the two groups (SHR: 154.4 ± 3.8 mmHg, SHR + L-NAME: 152.1 ± 3.9 mmHg, Figure 1).

Heart weight, kidney weight, and body weight and their relative ratio are shown in Table 1. There was a significant decrease in body weight after L-NAME treatment (*p* < 0.001). The heart weight/body weight as well as the kidney weight/body weight ratio was significantly increased in the L-NAME group (*p* < 0.001).

ROM, BAP, and TTL measurements from the plasma samples revealed no changes after chronic treatment with L-NAME (Table 1).

### 3.2. The Vasoactive Responses of the Thoracic Aorta

**Figure 2:**
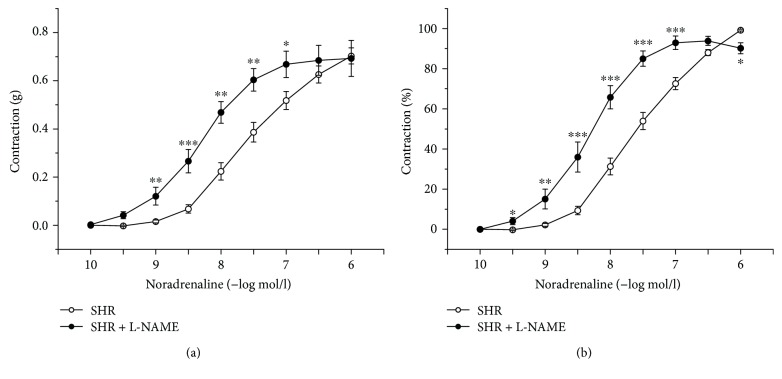
Concentration-response curves for exogenous noradrenaline expressed in grams (a) and as percent values of the maximum tissue response to noradrenaline (b). SHR: control group (*n* = 8); SHR + L-NAME: SHR treated with L-NAME (*n* = 7). Values are the mean ± S.E.M. ^∗^*p* < 0.05; ^∗∗^*p* < 0.01; ^∗∗∗^*p* < 0.001 versus SHR.

Chronic treatment with L-NAME significantly reduced the contractile response to KCl (*p* < 0.001) compared to the control SHR (data not shown). Cumulative application of noradrenaline (10^−10^–3 × 10^−5^ mol/l) induced vasoconstriction in a concentration-dependent manner. Although the dose-dependent curve of noradrenaline was shifted to the left (10^−9^–10^−7^ mol/l; *p* < 0.05, *p* < 0.01, and *p* < 0.001) in the L-NAME group (*n* = 7; Figure 2(a)), the maximal force of the contraction was not changed compared with the control SHR (*n* = 8). The changes in active tension correlated with alterations in sensitivity of the smooth muscle cells to exogenous noradrenaline. NOS inhibition by L-NAME significantly shifted the dose-dependent curve to the left; the curve is expressed as a percentage of the maximal vasoconstriction (*p* < 0.05, *p* < 0.01, and *p* < 0.001; Figure 2(b)). Moreover, EC_50_ values were significantly increased in the L-NAME group (8.44 ± 0.17, *p* < 0.01) compared to the untreated group (7.79 ± 0.06).

**Figure 3:**
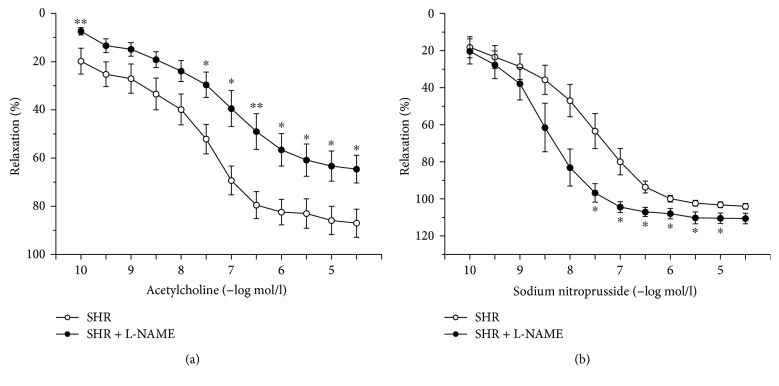
Endothelium-dependent relaxation evoked by acetylcholine (a) and endothelium-independent relaxation evoked by sodium nitroprusside (b) administration. SHR: control group (*n* = 8), SHR + L-NAME: SHR treated with L-NAME (*n* = 7). Values are the mean ± S.E.M. ^∗^*p* < 0.05, ^∗∗^*p* < 0.01 versus SHR.

The application of acetylcholine (10^−10^–3 × 10^−5^ mol/l) evoked a concentration-dependent vasorelaxation of the precontracted TA with phenylephrine (10^−6^ mol/l). Chronic treatment with L-NAME initiated a significant, but mild, inhibition of acetylcholine-induced vasorelaxation (*p* < 0.05, *p* < 0.01) compared with the control group (Figure 3(a)). While the maximal relaxant response in control SHR (*n* = 8) reached 87.02 ± 5.85%, the response reached 64.6 ± 5.75% in the L-NAME group (*n* = 7), which represented a surprisingly high percentage of vasorelaxation with regard to chronic inhibition of endogenous NO production by L-NAME. Our data showed no significant difference in IC_50_ values between the control SHR and L-NAME groups (SHR: 7.05 ± 0.29, SHR + L-NAME: 7.91 ± 0.39). The endothelium-independent vasorelaxation of the TA was induced by cumulative doses of the NO donor sodium nitroprusside (10^−10^–3 × 10^−5^ mol/l). Compared with the control group (*n* = 8), L-NAME treatment (*n* = 7) significantly increased the vasoactive response to exogenous NO; not only a significant difference of maximal reached vasorelaxation (*p* < 0.05, SHR: 104.02 ± 1.49%, SHR + L-NAME: 110.6 ± 2.88%) but also a shift of the dose-dependent curve to the left was observed (3 × 10^−8^–10^−5^ mol/l, Figure 3(b)).

**Figure 4:**
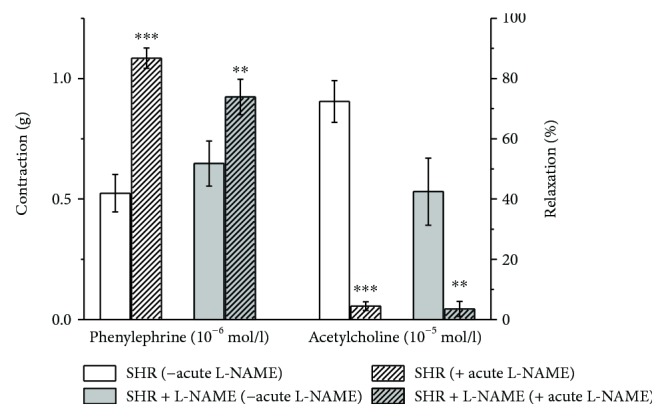
Effect of acute NO synthase inhibition induced by L-NAME (10^−4^ mol/l) on vasoactive responses induced by phenylephrine (10^−6^ mol/l) and acetylcholine (10^−5^ mol/l). SHR: control group (*n* = 8), SHR + L-NAME: SHR treated with L-NAME (*n* = 7). Values are the mean ± S.E.M. ^∗∗^*p* < 0.01; ^∗∗∗^*p* < 0.001 versus SHR.

Because the notable residual acetylcholine-induced vasorelaxation was recorded in the L-NAME group, the arteries from both groups were acutely pretreated with L-NAME (10^−4^ mol/l). We investigated the modulation effects of L-NAME on contractile responses induced by phenylephrine (10^−6^ mol/l) as well as on the vasorelaxation responses evoked by a maximal dose of acetylcholine (10^−5^ mol/l, Figure 4). The acute L-NAME pretreatment induced a significant increase in phenylephrine-induced contraction in both groups (*p* < 0.01, *p* < 0.001). Moreover, after acute pretreatment with L-NAME, we observed an inhibition of the vasorelaxation responses to acetylcholine not only in the control (*n* = 8, *p* < 0.001) but also in the L-NAME group (*n* = 7, *p* < 0.01). These results proved the presence of endogenous NO synthesis even after the long-term nonspecific NOS inhibition by L-NAME.

### 3.3. NO Synthase Activity

**Figure 5:**
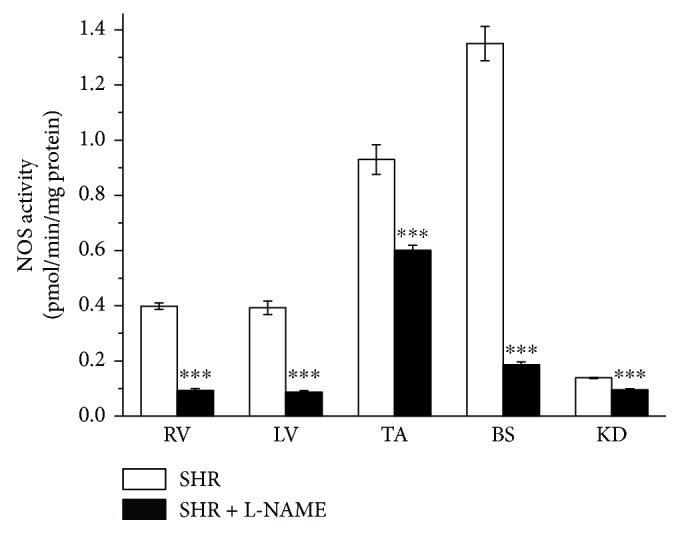
The evaluation of NO synthase activity in different tissues. RV: right ventricle; LV: left ventricle; TA: thoracic aorta; BS: brain stem; KD: kidney. SHR: control group (*n* = 8); SHR + L-NAME: SHR treated with L-NAME (*n* = 7). Values are the mean ± S.E.M. ^∗∗∗^*p* < 0.001 versus SHR.

Chronic treatment with L-NAME induced a significant decrease in NOS activity in all the monitored tissues. However, the degree of preserved NOS activity varied from tissue to tissue: from lowest in the brain stem to highest in the TA and in the kidney (Figure 5).

### 3.4. Detection of ROS Formation, Elastin Arrangement, and Wall Thickness in the TA

**Figure 6:**
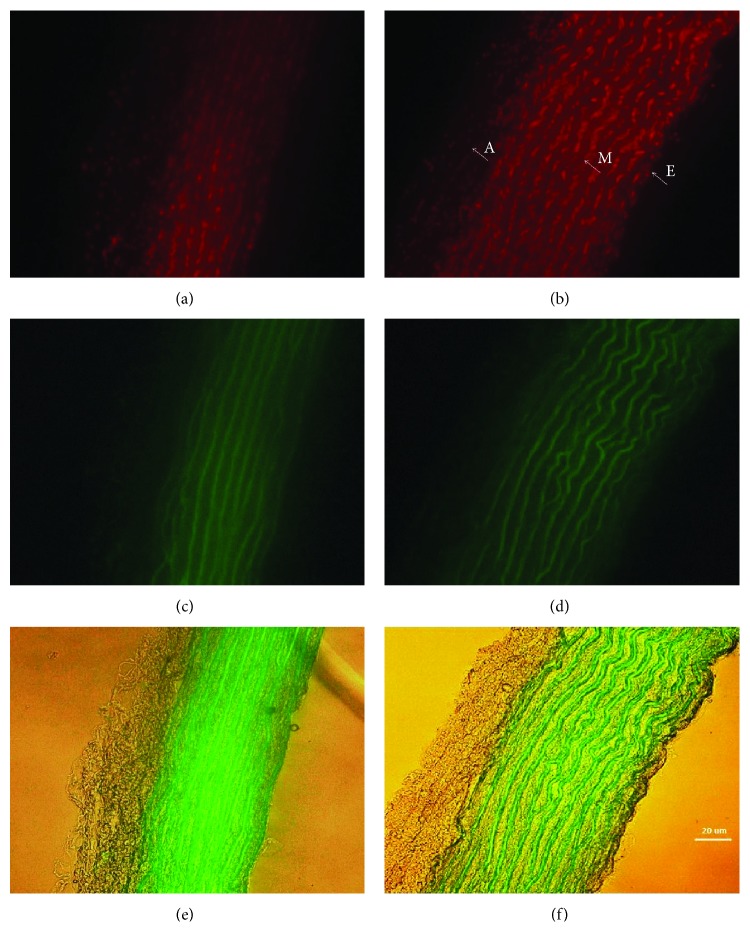
ROS production and structural changes (elastin arrangement and wall thickness) in the TA after L-NAME treatment in young SHR. DHE fluorescence detection (ex 540 nm/em 590 nm) was used to assess vascular redox homeostasis disruption in aortic cryosections, where ROS formation was determined by calculating the intensity (density/area) (a, b). Elastin autofluorescence (ex 488 nm/em 510 nm) was detected using fluorescence microscopy of aortic cryosections (c, d). Wall thickness: tunica intima + tunica media was determined in *μ*m (e, f). E: endothelium; M: tunica media; A: adventitia; SHR: control group (*n* = 8); SHR + L-NAME: SHR treated with L-NAME (*n* = 7).

L-NAME treatment significantly increased the formation of ROS by 20.8% (24.52 ± 0.771 int/area, *n* = 7) compared to the control group (20.3 ± 0.664 int/area, *n* = 8, *p* < 0.01, Figures 6(a) and 6(b)). Elastin is a highly elastic protein in the connective tissue that can resume its shape after stretching or contracting. Elastin arrangement was also altered after L-NAME treatment (Figures 6(c) and 6(d)). The area between elastin lines was increased, and few loops were observed in the tunica media and tunica intima in the L-NAME group, which could correlate with arterial wall hypertrophy: L-NAME-treatment induced 22.3% increase in wall thickness (70.63 ± 2758 *μ*m, *n* = 7) compared to the untreated group (58.75 ± 2.518 *μ*m, *n* = 8, *p* < 0.01; Figures 6(e) and 6(f)).

## 4. Discussion

Permanently increased sBP is developed gradually in SHR; in 3–5-week-old SHR, it does not differ from the age-matched normotensive rats. From 6 weeks of age, it becomes higher, and continual increasing of sBP is stopped after about 36 weeks of the age [15–17]. In this study, chronic L-NAME treatment in young SHR induced a transient increase in sBP in the 3rd week of treatment. In the 5th week of NO inhibition, there were no sBP differences in comparison with the control group, despite deteriorating health conditions, that is, disorientation, paralysis of lower extremities, and body weight loss. In adult SHR, Ono et al. [18] and Benter et al. [19] demonstrated that 2- to 4-week-long L-NAME treatment caused a sustained increase in malignant hypertension, that is, rapid weight loss and organ malformation. Several authors proved the existence of functional damage to the myocardium (fibrosis and cardiac infarction) and the kidney (proteinuria and nephrosclerosis) after L-NAME treatment, often with fatal results associated with myocardial infarction and cerebrovascular accidents [18]. In association with the morphological changes, the relative weight ratio of organs could also be altered. In our experiment, both the heart weight/body weight and the kidney weight/body weight ratios were significantly higher after L-NAME treatment. The observed hypertrophy could be attributed to lower body weight of the SHR, which is specific for this strain [9], and it was even more noticeable after the endogenous NO inhibition. However, we showed that the inhibition of NOS activity was not developed to the same degree in the tissues. A degree of preserved NOS activity varied between tissues: 13.7% in the brain stem, 22.1% in the left ventricle, 23.2% in the right ventricle, 64.6% in the thoracic aorta, and 68.4% in the kidneys. Our findings suggested that, in young SHR, the chronic inhibition of NO levels evoked negative and malignant changes. However, contrary to the case in adult rats, this occurred without developing a steadily increased sBP and a crucial decrease in NO levels in the cardiovascular system.

In the present study, we evaluated the vasoactive properties of the TA which represents an elastic type of the artery. Chronic L-NAME treatment significantly increased sensitivity of the adrenergic receptors; on the other hand, the maximal absolute contraction was not affected (Figures 2(a) and 2(b)). Moreover, we demonstrated that the vasoconstrictor response induced by depolarization of smooth muscle cells by KCl was significantly decreased. The treatment with L-NAME decreased or did not change the contractile power of smooth muscle cells in the TA. In our previous study, we demonstrated that, in young normotensive Wistar rats, continuous 6-week administration of L-NAME resulted in a reduced adrenergic contraction of the TA [20]. In young normotensive rats, the long-term NO blockage upregulated the endogenous vasoconstrictor system and probably triggered an adaptive mechanism: the downregulation of the contractile response to exogenous vasoconstrictors. Additionally, Oliver et al. [21] demonstrated that, after chronic inhibition of NO synthesis, adaptive mechanisms associated with diminished sensitivity to *α*-adrenergic vasoconstriction accompanied by increased *β*-adrenergic vasodilation occurred in the TA of normotensive WKY rats. On the other hand, Oliver et al. [22] demonstrated that in the TA of SHR the ratio of *α*-adrenergic and *β*-adrenergic receptors was changed and proved an age-correlated increased sensitivity of *α*-adrenergic receptors in 6- to 16-week-old SHR. Our finding of the increased TA sensitivity to noradrenaline after L-NAME treatment could be associated with this phenomenon and fits the age-dependent pathological changes of adrenergic receptors occurred in SHR.

In different normotensive rat models (Wistar and Sprague-Dawley), long-term NO deficiency revealed myocardial and arterial structural alterations such as necrosis, fibrosis, lipid deposition increased the production of extracellular matrix and aortic stiffening [23–26]. The changes in the arrangements of arterial wall components (collagen fibers, elastin filaments, and extracellular matrix) evoked by NO deficiency may compromise the contractile function of smooth muscle cells [27]. On the other hand, other studies confirmed that, in adult hypertensive NO-defective Wistar rats as well as SHR, the wall thickness of conduit arteries was increased, and an increased adrenergic contraction as a response of an increased smooth muscle mass of the arterial wall was followed [28–30]. In this study, we determined that the TA of L-NAME-treated animals underwent structural changes and the thickness of the arterial wall as well as the changes in the elastin arrangement was confirmed. It was accompanied by unchanged (noradrenaline) or decreased (KCl) absolute contractility and increased sensitivity of adrenergic receptors. Thus, the final contractile properties of the TA were formed by several mutual and/or contradictory processes. In young prehypertensive SHR, unlike in young Wistar rats, the chronic NO deficiency can modify the adaptive properties of the TA since the increased sensitivity to noradrenaline was observed. Hereby, unlike in adult SHR, the unincreased absolute force of the contractile responses is in a good consent with adaptive abilities of this vessel.

In this study, we confirmed 80% endothelium-dependent relaxation of the TA to acetylcholine in control SHR. The chronic inhibition of endogenously produced NO after L-NAME treatment stimulated only partial reduction of vasorelaxation; the inhibition of the maximal vasorelaxation response was surprisingly 35% (Figure 3(a)). Moreover, this finding was supported by biochemical measurement of NOS activity in the TA, which showed only 35.4% inhibition after L-NAME treatment (Figure 5). On the other hand, previous papers demonstrated that L-NAME treatment in adult SHR significantly inhibited endothelium-derived vasorelaxation [19, 31] and a similar reduction of acetylcholine-induced vasorelaxation after chronic L-NAME treatment was also demonstrated in adult and young normotensive rats [20, 32, 33]. To determine if preserved relaxation was mediated by endogenous NO, we acutely pretreated the TA with L-NAME (10^−4^ mol/l) (Figure 4). A significantly blocked relaxation response to acetylcholine and a potentiated contractile response to phenylephrine in the chronically treated L-NAME group suggested that there was a sufficient amount of physiologically active NO contributing to the maintenance of the relaxant responses, even after chronic nonspecific NOS inhibition. It also correlated with an unchanged sBP at the end of the treatment (blood pressure was increased only in the 3rd week). Several studies confirmed that conduit arteries in SHR could have undamaged endothelium-dependent vasorelaxation and enhanced endogenous production of NO [5]. Moreover, our previous study showed that NO contributed to the vasorelaxation responses of the TA in the prehypertensive SHR significantly more than in age-matched Wistar rats [8].Thus, we can suggest that in young SHR, the treatment with L-NAME was not able to fully inhibit the increased NO production in the TA. On the other hand, we might also assume that certain adaptive mechanisms could be triggered in young SHR. Since we also observed an increased maximal response as well as sensitivity to sodium nitroprusside after L-NAME administration, we may consider as a compensatory mechanism the increased sensitivity of smooth muscle cells to NO itself (Figure 3(b)). Moreover, recent study has proven that the prehypertensive phase of SHR is associated with an increased production and activity of asymmetric dimethyl L-arginine (ADMA), which is an endogenous inhibitor of NOS [34, 35]. Cheng et al. [36] demonstrated that continuous 6-week L-NAME treatment caused an additional increase in the plasma levels of ADMA in these rats. In the cardiovascular system, the principle of negative feedback regulation is often present. Kristek et al. [37] and Dovinova et al. [38] showed that, in SHR, an increased NO concentration may cause an inhibitory effect on NOS activity. Pechánová et al. [28] determined a negative correlation between endothelial NOS expression and activity: an increased expression resulted in an activity reduction in order to maintain a constant level of NO in the cardiovascular system. Cebova et al. [39] showed that although the long-term NO inhibition with L-NAME in adult WKY inhibited the NOS activity in the TA, it had no impact on the NOS protein expression. Regarding these facts, we speculated that only partially inhibited acetylcholine-induced vasorelaxation in the TA could be a negative feedback response of the cardiovascular system. If the level of ADMA as an endogenous NOS inhibitor is higher in young SHR, an addition of exogenous NOS inhibitor (L-NAME) could stimulate a compensatory (maybe tissue-specific) response and attenuate inhibition of NOS activity.

Kröller-Schön et al. [40] and Wenzel et al. [41] declared that, in the control (healthy) tissue, treatment with the NOS inhibitor (L-NAME) led to the inhibition of NO formation from intact NOS, resulting in the increased superoxide production. In the pathological stages, one of the mechanisms of the increased oxidative stress is the formation of uncoupled NOS, which produces superoxide instead of NO. Javkhedkar et al. [42], Bhatt et al. [3], and Chen et al. [43] confirmed the formation of uncoupled NOS in SHR, which subsequently led to the overproduction of superoxide. As such, the treatment with L-NAME induces the direct inhibition of uncoupled NOS, which leads to the decrease in superoxide production and oxidative stress in the diseased tissue [3, 40, 41]. Nevertheless, in our experiment, we determined that the chronic treatment with L-NAME induced an increase of ROS approximately 21% (and not a decrease) in the TA. It seems that the NOS enzyme in the TA of SHR is sufficiently intact to ensure a functional NO pathway. This suggestion that was also supported by the preserved (control group) or mildly inhibited (treated group) vasorelaxation. However, several mechanisms and pathways could be responsible for the production of free radicals in the cardiovascular system. In general, nicotinamide adenine dinucleotide phosphate oxidase (NOX) acts as the primary source of superoxide anions and H_2_O_2_ in the vessel wall. NOX-derived ROS then act as “kindling” and activate secondary (uncoupled eNOS, xanthine oxidase) and tertiary (mitochondrial) sources of ROS, which contribute to the “bonfire” of radicals and oxidative stress seen at later stages of diseases [44]. An increased NOX activity can be influenced by NO production. It has been showed that endogenous NO attenuated NADPH oxidase-dependent superoxide production and that after L-NAME treatment a significant increase in aortic p47phox (a main regulator of NOX activation expression) was observed [45]. So it seems that vascular oxidative stress might be suppressed by NO donors and, on the other hand, the decreased NO levels could increase NADPH oxidase activation in vascular tissue. ROS have a very short half-life and cannot be measured in plasma or serum samples. Therefore, derivatives of reactive oxygen metabolites (d-ROM) as a proxy for ROS production and total thiol levels (TTL) of proteins in serum as a proxy for the redox control status were measured [46]. BAP test provides a useful assessment of antioxidant activity of blood plasma. However, our results showed that after L-NAME treatment, there were no differences in ROM, TTL, and BAP. Therefore, it seems that chronic NO deficiency in young SHR probably did not lead to disorder in general redox status, and followed functional malfunctions could rather be a cause of tissue-specific impairment.

Our findings proved that chronic NO deficiency, initiated in the prehypertensive period of the SHR, led to the development of adaptive vasoactive mechanisms. TA of young SHR responded to chronic L-NAME treatment by the development of adaptive mechanisms on the functional (preserved NO-derived vasorelaxation, unincreased contraction) and molecular (preserved NOS activity) level. Our findings also confirmed the structural remodeling (wall thickness, changes in elastin arrangement) and increased superoxide production in the TA; however, the selected biomarkers of the oxidative stress in plasma remained unchanged.

## Figures and Tables

**Table 1:** General cardiovascular parameters and biomarkers of oxidative stress in young SHR after chronic NO synthase inhibition.

	*n*	BW (g)	HW (mg)	HB (mg/g)	KW (mg)	KB (mg/g)	ROM (IU/l)	TTL (*μ*mol/l)	BAP (meq/l)
SHR	8	248 ± 9.8	995.5 ± 42.3	4.0 ± 0.1	1829.7 ± 75.4	7.4 ± 0.1	297 ± 5	323 ± 15	2619 ± 37
SHR + L-NAME	7	180.4 ± 9.1^∗∗∗^	1026.3 ± 110.0	5.3 ± 0.1^∗∗∗^	1593.4 ± 99.7	8.8 ± 0.2^∗∗∗^	314 ± 27	278 ± 18	2708 ± 130

*n*: number of rats; BW: body weight; HW: heart weight; HB: heart weight/body weight ratio; KW: kidney weight; KB: kidney weight/body weight ratio; ROM: reactive oxygen metabolites; TTL: total thiol levels; BAP: biological antioxidant potency. Values are the mean ± S.E.M. ^∗∗∗^ *p* < 0.001 versus SHR.

## Data Availability

All data arising from this study are contained within the article and any additional data sharing will be considered by the first author upon request.
